# Occurrence of Multidrug-Resistant and Toxic-Metal Tolerant Enterococci in Fresh Feces from Urban Pigeons in Brazil

**DOI:** 10.1264/jsme2.ME11296

**Published:** 2011-12-22

**Authors:** Vânia Lúcia da Silva, Natália Cândido Caçador, Carolina dos Santos Fernandes da Silva, Cláudia Oliveira Fontes, Gizele Duarte Garcia, Jacques Robert Nicoli, Cláudio Galuppo Diniz

**Affiliations:** 1 Department of Parasitology, Microbiology and Immunology, Institute of Biological Sciences, Federal University of Juiz de Fora, Juiz de Fora, Brazil; 2 Department of Genetics, Institute of Biology, Federal University of Rio de Janeiro, Rio de Janeiro, Brazil; 3 Department of Microbiology, Institute of Biological Sciences, Federal University of Minas Gerais, Belo Horizonte, Minas Gerais, Brazil

**Keywords:** pigeon feces, enterococci, antimicrobial drugs, toxic metals

## Abstract

*Enterococcus* are emerging as important putative pathogens resistant to chemicals that are widely released into the environment, and urban pigeons might act as a natural reservoir contributing to the spread of resistant strains. This study aimed to evaluate the occurrence of *Enterococcus* in pigeon feces and their antimicrobial and toxic metal susceptibility. Bacteria were isolated and identified from 150 fresh feces by phenotypic and genetic techniques. Antimicrobial and toxic metal susceptibility was determined by the agar dilution method, and the multiple antibiotic resistance index (MAR) was calculated. Out of 120 isolates, no resistance was observed against penicillin and vancomycin, but was observed against gentamicin (55.8%), chloramphenicol (21.7%), tetracycline (13.3%), ciprofloxacin (8.4%) and rifampin (2.5%). 18.3% presented a MAR index ≥0.2, ranging between 0.14 to 0.57, indicating resistance to more than one antimicrobial. All samples were tolerant to >1024 μg mL^−1^ zinc and chromium. Minimal inhibitory concentration (MIC) of 1,024 μg mL^−1^ was observed for copper (100%) and nickel (71.4%). Mercury inhibited 88.4% at 32 μg mL^−1^ and the MIC for cadmium ranged from 0.125–128 μg mL^−1^. Since pigeons were found to harbor drug-resistant *Enterococcus*, our data support that their presence in the urban environment may contribute to the spread of resistance, with an impact on public health.

Urban pigeons (*Columba livia*) are birds that coexist with humans, especially in urban environments. It is accepted that their adaption to these environments is mainly related to three basic reasons: (i) the architecture of urban buildings, monuments and works of engineering has gaps, cracks and spaces that can be used for landing, nesting and shelter, protecting the birds from the weather; (ii) the absence of natural predators; and (iii) availability of food, as these birds are omnivorous, somewhat selective in their feeding (23). In this way pigeons may have an important role in spreading zoonotic agents to humans as they are a natural reservoir of many microorganisms, including pathogenic enterobacteria and other microbial groups such as *Enterococcus*(6, 19, 41). The transmission of infectious diseases happens usually by either ingestion of food, water, the handling of products contaminated with their feces. The dispersion of wind-dried pigeon droppings should also be considered as a route of pathogen spread (29, 36).

Enterococci are Gram-positive, facultative anaerobes, arranged in pairs and short chains. These bacteria were considered as harmless inhabitants of the gastrointestinal tracts of humans and animals, being commonly isolated from feces, water, plants, soil and food products (14, 33). Despite their low virulence, studies have documented that enterococci have been emerging as significant human pathogens (21, 25). Due to their frequent association with infections and their increasing antimicrobial resistance, enterococci have become a serious problem in human medicine as a pathogenic microorganism, as well as nosocomial infection-associated bacteria. Besides natural antimicrobial resistance among *Enterococcus* species to several antibiotics currently used, they may also acquire resistance determinants through horizontal gene transfer (5).

Furthermore, especially regarding bacteria spread environmentally, the release of toxic and heavy metals in various forms into the environment may contribute to the rise of antimicrobial resistance. In the last decade it was stated that antibiotic-resistant bacteria could arise in the environment through co- or cross-resistance to metals (30).

Considering global issues, such as public health, urban planning and environmental problems, as pigeon populations increase, especially in Brazilian cities, prospective studies are needed to better assess the risks of acquired infections related to multidrug-resistant bacteria that might be spread or released by urban birds (29, 41, 44). This study aimed to evaluate *Enterococcus* spp. occurrence in fresh fecal samples isolated from urban pigeons in a southeastern Brazilian city, their antimicrobial susceptibility and their toxic metal tolerance patterns.

## Material and Methods

### Isolation of *Enterococcus* spp. from pigeon feces

One hundred fifty fresh fecal samples were collected from urban pigeons at different sites in the metropolitan area of the city of Juiz de Fora, Brazil, between May 2007 and May 2008. As the pigeons were fed, fresh feces were collected with sterilized wooden sticks and placed in glass tubes containing sterile phosphate-buffered saline. Bacterial strains representative of different colonial morpho-types were isolated after selective culture in Bile Esculin Agar (Himedia Laboratories, Mumbai, India) supplemented with 0.01% (w/v) sodium azide (Vetec, Rio de Janeiro, Brazil). Presumptive phenotypic identification included esculin hydrolysis, a catalase test and Gram stain.

### Bacterial genus confirmation and species-specific identification

DNA from the presumptively identified bacteria was extracted by chemical digestion with phenol-chloroform, with some modifications (18). Briefly, bacteria cells from 2 mL overnight cultures (approximately 10^8^ CFU) in Brain Heart Infusion broth (Himedia Laboratories) were pelleted by centrifugation and solubilized in 500 μL bacterial lysis buffer (25% [w/v] sucrose, in 50 mmol L^−1^ Tris-HCl [pH 8.0], 10 mmol L^−1^ EDTA, 2.5 mg mL^−1^ lysozyme). The culture was incubated at 37°C for 60 min. Further, 50 μL of 20% (w/v) SDS solution was added and the culture was left at room temperature for 30 min. DNA was extracted three times with equal volumes of phenol and chloroform, followed by ethanol precipitation and RNAse treatment (10 μg mL^−1^; Sigma-Aldrich, St. Louis, MO, USA). DNA extracts were stored at −20°C until use.

*Enterococcus* genus identification was carried out by PCR amplification of 16S ribosomal RNA encoding DNA, as previously described (46), with the primers ENT1 (5′-AGGGGATAACACT TGGAAACA-3′) and ENT2 (5′-TTCGCGACTCGTTGTACTTC-3′). The reactions (25 μL) consisted of 5 ρmol L^−1^ of each primer, 2 μL template DNA and 12.5 μL of a ready-to-use 2X PCR Master Mix (Promega Corporation, Madison, WI, USA), with the following amplification conditions: 95°C, 5 min, 30 cycles of 95°C, 30 s, 65°C, 30 s, 72°C, 30 s, and 72°C, 5 min. All the PCR reactions were performed in duplicate in a thermal cycler (Techne TC-412; Southam, Warwickshire, UK). The expected amplicons (1,178 bp) were observed after 1% (w/v) agarose gel electrophoresis and the reference strain *Enterococcus faecalis* ATCC 51299 was used as a positive control.

Species identification was performed by multiplex PCR as previously described (22), with the exception of *E. faecalis* and *Enterococcus faecium*, for which standard PCR was performed (24, 43). The primers and PCR conditions are described in Table 1. Reactions were performed in a 25 μL mixture containing 2.0 μL DNA template, primers at a final concentration of 16 μmol L^−1^ and 12.5 μL of ready-to-use 2X Qiagen PCR Multiplex Master Mix (Qiagen, Hilden, Germany) for multiplex reactions or ready-to-use 2X PCR Master Mix (Promega) for standard PCR. The reactions were performed in an automated thermal cycler (Techne TC-412) and amplicons were observed after 2% (w/v) agarose gel electrophoresis.

### Antimicrobial susceptibility assays

The minimum inhibitory concentrations (MIC) for antimicrobial drugs and toxic metals were determined by the agar dilution method, according to the Clinical and Laboratory Standard Institute guideline (11). Antibiotic or toxic metal stock solutions were added to melted Muller-Hinton (HiMedia) agar to obtain final concentrations ranging from 0.06 to 1,024 μg mL^−1^. The antimicrobial drugs were selected on the basis of microbial characteristics and clinical relevance: penicillin, gentamicin, vancomycin, chloramphenicol, rifampin, tetracycline and ciprofloxacin (all MedQuimica, Minas Gerais, Brazil). The reference strains *E. faecalis* ATCC 51299 and *Staphylococcus aureus* ATCC 29213 were included as controls and all tests were performed in duplicate. To determine the level of antibiotic resistance of the individual isolated bacteria, the multiple antibiotic resistance (MAR) index was calculated according to the literature (26), by dividing the number of antibiotics to which the isolate was resistant by the total number of antibiotics to which the isolates were exposed. A MAR value >0.2 was indicative of multiple antibiotic-resistant bacteria.

Toxic metals were selected on the basis of their environmental availability and biologic relevance: nickel (NiCl_2_·6H_2_O), zinc (ZnSO_4_·7H_2_O), mercury (HgCl_2_), cadmium (CdCl_2_·H_2_O), chromium (Cr(NO_3_)_3_) and copper (CuSO_4_) (all Vetec). The reference strains *E. faecalis* ATCC 51299, *S. aureus* ATCC 29213 and *Escherichia coli* ATCC 25922 were used as a control. All tests were performed in duplicate. The MIC was determined as the lowest concentration of metal salt that completely inhibited bacterial growth. The results are expressed as the level of tolerance based on toxic concentrations of these substances to other biological systems and compared to the reference values of MIC for the strain *E. coli* K-12, according to the literature (3, 31).

## Results

Out of 150 fecal samples isolated from 150 pigeons in different urban areas of Juiz de Fora, Brazil, 175 strains of Gram-positive cocci presumptively identified as enterococci were recovered and 120 had identity confirmation to the genus level based on the amplification of 16S ribosomal RNA encoding DNA.

Regarding species-specific identification of *Enterococcus*, four different species were identified: *E. faecium* (2, 1.66%), *E. faecalis* (5, 4.16%), *Enterococcus solitarius* (13, 10.83%) and *Enterococcus columbae* (81, 67.5%); however, according to the methodology, 19 isolates (15.83%) had no specific identification and were assigned as *Enterococcus* sp.

The results of the antimicrobial drug susceptibility assays are shown in Table 2, and are presented in terms of MIC_50_ (lowest concentration that inhibits 50% of bacterial population), MIC_90_ (lowest concentration that inhibits 90% of bacterial population) and the range of MICs. The antimicrobial susceptibility patterns for the control strains *E. faecalis* ATCC 51299 and *S. aureus* ATCC 29213 were in accordance with CLSI. Penicillin and vancomycin were the most effective drugs, and all enterococci strains were susceptible. Considering all 120 strains, resistance was observed against chloramphenicol (26, 21.7%), tetracycline (16, 13.3%), ciprofloxacin (10, 8.4%) and rifampin (3, 2.5%). Gentamicin was the least effective drug, and high-level gentamicin resistance was observed for 55.8% of enterococci strains.

Out of 120 evaluated strains, only 21.7% (*n=*26) were susceptible to all tested drugs, whereas 78.3% (*n=*94) were resistant to at least one antimicrobial drug. Resistance to more than one antimicrobial drug was observed, and the distribution of antimicrobial drug resistance phenotypes among *Enterococcus* strains is shown in Table 2. Regarding multi-drug resistance, 22 isolates (18.3%) showed MAR index ≥0.2, which is designated as multiple antimicrobial resistant. Isolates showed variations of the MAR index from 0.14 to 0.57. The species with the highest MAR index was *E. faecalis* (0.57), which showed simultaneous resistance to four antimicrobials.

As there are no standards for toxic metal susceptibility, we performed the tests according to the CLSI guidelines for antimicrobial drugs (11), and the results were referred to having high or low tolerance to toxic metals. All tested bacteria were tolerant to nickel, copper, zinc and chromium. No cadmium tolerance was observed, but most of the *Enterococcus* strains were tolerant to mercury, with the exception of 11 *E. columbae*. Among these mercury-susceptible bacteria, 5 strains were resistant only to gentamicin and 6 were susceptible to all tested antimicrobial drugs (Table 3).

Overall, all samples were highly tolerant to >1,024 μg mL^−1^ ZnSO_4_ and Cr(NO_3_)_3_. MIC of 1,024 μg mL^−1^ was observed for CuSO_4_ (100%) and NiCl_2_ (71.4%). Regarding HgCl_2_, 88.4% of isolates were inhibited at 32 μg mL^−1^ and the lowest tolerance was observed against CdCl_2_, with MIC ranging from 0.125–128 μg mL^−1^, with MIC_50_ 8 μg mL^−1^, as observed in Table 4.

## Discussion

The potential spread of infectious agents from animals to humans is high, and this is particularly pronounced in urban birds (20). The food chain may be one way of determining how bacteria are being spread, but environmental pollution is also important (10). In this regard, pigeons could play an important role in fecal contamination of drinking water sources and agricultural crops surrounding urban areas and might also come into close contact with domestic animals, enabling direct transfer of infectious agents. *C. livia* might also favor the inter-genus or inter-species horizontal spread of drug-resistance genetic determinants, because their feces can harbor several infectious agents representative of different environments that the birds recently visited (10, 20). Since these pigeons have peculiar gut microbiota, it is possible that these microorganisms are not true inhabitants of this ecosystem. The intestinal microbiota of pigeons probably differ from those of gallinaceous species, such as chickens. Pigeons possess a less developed cecum, which is the part of the gastrointestinal tract that harbors the most abundant and diversified microbiota. As a result, it has been suggested that these birds could not have a permanent gut microbiota with intestinal bacterial species commonly associated with humans (20, 41). The feeding habits of urban pigeons could enable them to be contaminated with medically important bacteria or residual antimicrobials and chemicals, since they may rely on garbage or nearby trash cans as food sources (37). Considering the geography of the city of Juiz de Fora, it is hard to address the origin of these urban pigeons. Indeed there are several nosocomial areas as well as public gardens and squares spread around the city, no different from other large metropolitan areas in the world.

*Enterococcus* species naturally associated with humans are rarely found in pigeons; however, their dietary habits may cause them to be contaminated with different bacterial species, chemical residues and antibiotics (6, 12, 23). Indeed, in our study, we observed not only the presence of *E. faecium* and *E. faecalis* in pigeon feces, which are human-related species, but other *Enterococcus* species showing drug resistance and toxic metal tolerance.

Enterococci infections have become increasingly common due to their intrinsic resistance to several antimicrobial agents and their propensity to acquire resistance genetic determinants from the environment. In this study, the isolates were phenotypically tested for antimicrobial resistance, but no molecular investigation of genes encoding antimicrobial resistance was carried out. In this way, it is difficult to address the origins of drug-resistance determinants or even to track the spread of bacterial strains from the nosocomial environment. None of the isolated bacteria were resistant to penicillin and vancomycin, in agreement with scientific information available from Europe regarding *E. faecalis* isolated from pigs, chickens, humans and wild rabbits (1, 2, 38, 42). However, in Brazil there are few reports of low β-lactam resistance in enterococci isolated from humans and animal products (8, 13, 16). The observed susceptibility to penicillins and glycopeptides might be a good outcome, since between these antimicrobial substances there are several compounds used to treat severe enterococci-related infections (8, 13). Considering vancomycin, we also did not observe any resistant strain, although for *E. faecium* isolated from chickens and pigs in Europe, resistance was observed at rates as high as 17% (1, 2). Non-human related enterococci resistance to vancomycin was also reported in Australia where *Enterococcus gallinarum* and *Enterococcus casseliflavus* were isolated from the intestinal tracts of slaughter pigs (15).

Glycopeptides are used as the last treatment option for severe nosocomial enterococci-related infections of immuno-compromised patients, once penicillins prove to be ineffective. Resistance to vancomycin among *Enterococcus* in Brazil has been mostly associated with *E. faecalis*, contrasting with data from the United States and Europe which have identified *E. faecium* as the most common vancomycin-resistant enterococci. The lack of community reservoirs of vancomycin-resistant *Enterococcus* in Brazil also contrasts with epidemiological findings from Europe (28, 40).

Regarding high-level concentrations of gentamicin (an aminoglycoside), 55.8% of *Enterococcus* strains isolated in our study were resistant. Fard *et al.*(15) found susceptibility to high-level gentamicin for enterococci isolated from pigs; however, especially concerning human isolates, the ineffectiveness of this antibiotic was observed near to 20% (8, 13). With regards to animal origin and high-level gentamicin-resistant enterococci, Silva *et al.*(42) observed low rates of 6.3% among bacteria recovered from wild European rabbit fecal samples.

High-level aminoglycoside resistance (HLAR) was first reported in 1979; thereafter, increasing occurrence has been documented in hospitals worldwide. In previous studies with enterococci isolated in Brazil, the overall prevalence of HLAR among clinical isolates showed a tendency to increase over the years (32). In Brazil, β-lactam in association with gentamicin is one of the first choice drugs to treat severe enterococcal infections (45).

Fluoroquinolones are effective in treating a wide variety of infections and are the antimicrobials of choice for treating invasive gastrointestinal infections in adult humans in many parts of the world. These drugs may also be used to treat enterococal urinary tract infections and it is accepted that urban pigeons may play an important role in spreading quinolone-resistant bacteria, such as *E. coli* in urban, suburban and agriculture environments (39).

Considering the resistance to ciprofloxacin observed in this study (8.4%), similar rates have been recorded for *Enterococcus* strains isolated from wild animals worldwide (8.6%), although among bacteria isolated from wild European rabbit fecal samples and humans, the resistance rates are higher as of 14.1% and 30%, respectively (8, 13, 16, 38, 42).

As rifampin remains widely used in Brazil, and in addition to the 2.5% resistance observed, our results show lower resistance rates if compared to other national studies of enterococci isolated from hospitalized patients, which showed resistance rates of about 24% (8). In the same way, regarding tetracyclines, as these drugs are still used as food supplements or therapy in poultry and young animals, the resistance rates observed in this study, considering urban birds (13.3%), were lower than reported in different regions of the globe, including enterococci strains isolated from different animals and other environmental sources (2, 8, 13, 15, 16, 38, 42). Although tetracycline is not routinely used to treat enterococal infections, resistance to this antimicrobial agent is still common among clinical- and environmental-associated strains (27).

According to the literature, chloramphenicol is a broad spectrum antimicrobial largely used in the treatment of nosocomial infections, and enterococci are among the most chloramphenicol-resistant bacteria of human origin, with resistance rates as high as 33% (1, 8, 13, 17). In this study, the resistance observed against this antimicrobial (21.7%) was higher than reported for *Enterococcus* spp. isolated from other animals (rates of up to 7%), with the exception of some strains isolated from pigs in Spain, which showed resistance rates of 19% (2, 16, 38).

Overall, the frequency of MAR >0.2 observed in our study among the bacteria isolated alerted us to the possible role of urban pigeons as potential agents for the spread of resistance genes. These multi-resistant bacteria may reflect the microbial adaptive response to residual antimicrobials and chemicals in the environment, since pigeons could rely on garbage and also can be found in nosocomial areas.

Resistance to antimicrobials, including toxic metals, is important for bacteria survival in contaminated environments. In most of the times, several antibiotic and heavy metal resistance genes might share the same mobile genetic elements, such as plasmids, transposons and integrons (21). As a biologic consequence, these genes might be exchanged among bacteria living in highly contaminated areas. It has been suggested that the combination of antibiotic and metal resistance may not be a fortuitous phenomenon, and bacterial resistance against toxic metals appears to be directly related to the presence of these elements as environmental pollutants; therefore, it is suggested that the natural selective pressure imposed by toxic metals may indirectly select for resistance to antimicrobial drugs (5, 25).

In our study, all enterococci were tolerant to zinc, chromium, nickel and copper. Copper and zinc are extensively used in animals as growth-promoting additives (pigs, livestock, cattle, etc), and that urban pigeons may have contact with these agents through their dietary habits and, in this way, several bacteria species developed resistance mechanisms to survive copper toxicity (4). Copper tolerance, according to the literature, is usually mediated by a plasmid-born *tcrB* gene, and has been reported in *E. faecium* and *E. faecalis* isolated from pigs (2, 15). As for observations for zinc, copper tolerance and antimicrobial resistance have been reported (35).

With regards to mercury and cadmium, we observed low tolerance rates among the isolated enterococci, although mercury resistance is a common plasmid-mediated ability in both Gram-negative and Gram-positive bacteria (47). This phenomenon might be related to the use of mercurial compounds in industry, agriculture and hospitals. As for Gram-positive bacteria, including *E. faecalis*, mercury resistance is associated with antibiotic resistance plasmids, particularly those encoding β-lactamases (47). As we did not observe resistance to β-lactam drugs, this is reasonable to explain the low mercury tolerance observed in our study. There is little information available in the literature about enterococci tolerance to cadmium, especially considering bacteria isolated from non-human sources. In general, when reported, no high tolerance has been observed (34).

Different studies have reported toxic metal tolerance among bacteria isolated from diverse environments (31); however, there is a lack of technical standards in these experimental designs, resulting in difficulties for data comparison. To avoid this lack of technical standards, experiments for testing toxic metal susceptibility patterns among the isolated bacteria were performed by the agar dilution technique, as recommended for antimicrobial susceptibility testing by the Clinical Laboratory Standards Institute (11).

Thus, it is not surprising that toxic metal resistance genes are commonly found in environmental bacteria and that these genes may confer co-resistance or cross-resistance to antimicrobial drugs (7, 31). These co-selection mechanisms include co-resistance, *i.e.*, different resistance determinants present on the same genetic element such as a plasmid, and cross-resistance, *i.e.*, a single phenotype may be associated with both antimicrobial and metal resistance, such as an active efflux pump (7). In this way, the higher rates of toxic metal tolerance at high levels detected in most of the bacterial strains isolated in this study may be the result of toxic metal contamination in the environment. Moreover, it could be a consequence of the use of toxic metals in the urban and surrounding rural environments for water treatment, industry, agriculture and hospital disinfection purposes, among others uses (9).

Despite no correlation between antimicrobial resistance and toxic metal tolerance among the isolated *Enterococcus* strains, the high toxic metal tolerance patterns observed point to the high selective pressure to which bacterial populations associated to urban pigeons are exposed and the risks of the spread of genetic determinants which might confer, as stated previously, antimicrobial cross-resistance phenotypes.

Overall, in this study, antimicrobial resistance was observed to some antimicrobials that are important for human chemotherapy, especially in susceptible hosts. Such a phenomenon among other opportunistic *Enterococcus* spp. isolated from fresh urban pigeon feces hugely increases the potential of these birds to act as a reservoir of antimicrobial-resistant bacteria and/or -resistant genes that may be transferred to pathogens through the environmental chain. Monitoring of antibiotic resistance and toxic-metal tolerance in bacteria from environmental sources, such as urban pigeon feces, would help to identify relevant factors that contribute to the spread of resistant bacteria and would support the prudent use of antibiotics and discharge or final disposal of metal-contaminated residues.

## Figures and Tables

**Table 1:** Primers used for PCR reactions, sequences, amplicon size and amplification conditions

PCR Reaction	Primers	Species	Primer sequence (5′ to 3′)	Amplicon	Amplification conditions
Reaction I (multiplex)	CA1	*E. casseliflavus*	TCCTGAATTAGGTGAAAAAAC	288	95°C, 4 min; 30×95°C, 30 s; 55°C, 1 min; 72°C 1 min; 72°C, 7 min.
	CA2		GCTAGTTTACCGTCTTTAACG		
	GA1	*E. gallinarum*	TTACTTGCTGATTTTGATTCG	173	
	GA2		TGAATTCTTCTTTGAAATCAG		
	SO1	*E. solitarius*	AAACACCATAACACTTATGTGACG	371	
	SO2		AATGGAGAATCTTGGTTTGGCGTC		
	AV1	*E. avium*	GCTGCGATTGAAAAATATCCG	368	
	AV2		AAGCCAATGATCGGTGTTTTT		
	CO1	*E. columbae*	GAATTTGGTACCAAGACAGTT	284	
	CO2		GCTAATTTACCGTTATCGACT		
	SE1	*E. seriolicida*	ACACAATGTTCTGGGAATGGC	100	
	SE2		AAGTCGTCAAATGAACCAAAA		

Reaction II	EF1	*E. faecalis*	GTTTATGCCGCATGGCATAAGAG	310	95°C, 2 min; 26×95°C, 30 s; 60°C, 1 min; 72°C, 1 min; 72°C, 2 min.
	EF2		CCGTCAGGGGACGTTCAG		

Reaction III	EFA1	*E. faecium*	TTGAGGCAGACCAGATTGACG	658	94°C, 5 min; 30×94°C, 1 min; 54°C, 1 min; 72°, 1 min; 72°C, 10 min.
	EFA2		TATGACAGCGACTCCGATTCC		

**Table 2:** Susceptibility patterns of *Enterococcus* spp. (*n*=120) isolated from urban pigeon (*Columba livia*) feces in Juiz de Fora, Brazil

Tested antimicrobial drugs	Minimal Inhibitory Concentration (μg mL^−1^)			Susceptible strains (%)

	MIC_50_	MIC_90_	Range	
Penicilin	1	4	0.06–8	100.0
Ciprofloxacin	0.48	1	0.06–8	91.6
Rifampin	0.48	4	0.06–32	97.5
Tetracycline	0.48	64	0.12–>512	86.7
Chloramphenicol	8	16	4–16	78.3
Vancomycin	1	1	0.25–4	100.0
Gentamicin	512	512	—	44.2

MIC_50_: lowest antimicrobial concentration that inhibits 50% of the tested bacterial population;

MIC_90_: lowest antimicrobial concentration that inhibits 90% of the tested bacterial population.

**Table 3:** Distribution of antimicrobial drug resistance and metal tolerance phenotypes among *Enterococcus* spp. (*n*=120) isolated from urban pigeon (*Columba livia*) feces in Juiz de Fora, Brazil

Bacterial samples (*n*)	Phenotype		Frequency	

	Antimicrobial resistance	Metal tolerance	n	%
*Enterococcus columbae*(81)	GEN	Ni, Cu, Zn, Cr	5	6.17
	GEN	Ni, Cu, Zn, Hg, Cr	33	40.74
	CLO	Ni, Cu, Zn, Hg, Cr	22	27.16
	GEN, TET	Ni, Cu, Zn, Hg, Cr	7	8.64
	CLO, CIP	Ni, Cu, Zn, Hg, Cr	1	1.23
	*Susceptible to all drugs*	Ni, Cu, Zn, Cr	6	7.41
	*Susceptible to all drugs*	Ni, Cu, Zn, Hg, Cr	7	8.64

*Enterococcus faecalis*(5)	GEN, TET, CIP	Ni, Cu, Zn, Hg, Cr	1	20.0
	GEN, TET, RIF, CIP	Ni, Cu, Zn, Hg, Cr	1	20.0
	*Susceptible to all drugs*	Ni, Cu, Zn, Hg, Cr	3	60.0

*Enterococcus faecium*(2)	GEN	Ni, Cu, Zn, Hg, Cr	1	50.0
	GEN, TET	Ni, Cu, Zn, Hg, Cr	1	50.0

*Enterococcus solitarius*(13)	GEN	Ni, Cu, Zn, Hg, Cr	4	30.76
	GEN, CIP	Ni, Cu, Zn, Hg, Cr	5	38.46
	GEN, RIF	Ni, Cu, Zn, Hg, Cr	1	7.69
	GEN, RIF, CIP	Ni, Cu, Zn, Hg, Cr	1	7.69
	GEN, TET, CIP	Ni, Cu, Zn, Hg, Cr	1	7.69
	*Susceptible to all drugs*	Ni, Cu, Zn, Hg, Cr	1	7.69

*Enterococcus* sp. (19)	CLO	Ni, Cu, Zn, Hg, Cr	3	15.79
	TET	Ni, Cu, Zn, Hg, Cr	1	5.27
	GEN	Ni, Cu, Zn, Hg, Cr	3	15.79
	GEN, TET	Ni, Cu, Zn, Hg, Cr	3	15.79
	*Susceptible to all drugs*	Ni, Cu, Zn, Hg, Cr	9	47.36

The break point values for antimicrobial resistance were in agreement with CLSI, 2007 (11). RIF: rifampin; CLO: chloramphenicol; TET: tetracycline; CIP: ciprofloxacin; GEN: gentamicin. The break point values for metal tolerance were: 12.5 μg mL^−1^ for mercury (Hg), 100 μg mL^−1^ for cadmium (Cd), 200 μg mL^−1^ for nickel (Ni), 200 μg mL^−1^ for copper (Cu), 200 μg mL^−1^ for zinc (Zn) and 800 μg mL^−1^ for chromium (Cr) (3, 31).

**Table 4:** Metal tolerance patterns of *Enterococcus* spp. (*n*=120) isolated from urban pigeon (*Columba livia*) feces in Juiz de Fora, Brazil.

Tested toxic metals	Minimal Inhibitory Concentration (μg mL^−1^)		

	MIC_50_	MIC_90_	Range
Cadmium (CdCl_2_·H_2_O)	8	32	0.125–128
Copper (CuSO_4_)	1,024	1,024	—
Chromium (Cr(NO_3_)_3_)	>1,024	>1,024	—
Mercury (HgCl_2_)	32	32	8–128
Nickel (NiCl_2_·6H_2_O)	1,024	1,024	256–>1,024
Zinc (ZnSO_4_·7H_2_O)	>1,024	>1,024	—

MIC_50_: lowest antimicrobial concentration that inhibits 50% of the tested bacterial population;

MIC_90_: lowest antimicrobial concentration that inhibits 90% of the tested bacterial population.
